# Ten-year fall in blood cholesterol of Malaysia heart attack patients suggests statin impact

**Published:** 2018

A 10-year decline in the blood cholesterol of heart attack patients in Malaysia suggests that statins are having a positive impact, according to an observational study in nearly 49 000 patients presented at the ASEAN Federation of Cardiology Congress 2017 (AFCC2017).

AFCC2017 was hosted by the Brunei Cardiac Society, with the support of the ASEAN Federation of Cardiology, on 3 to 5 November in Brunei Darussalam. Experts from the European Society of Cardiology (ESC) presented a special programme.

‘Lifestyle changes appear to be responsible for falls in blood cholesterol in the general populations of developed nations while statins have reduced cholesterol in patients with heart disease,’ said lead author Dr Sazzli Kasim, Chair, Malaysian Society of Atherosclerosis and Associate Professor of Medicine, University Technology MARA, Shah Alam, Malaysia.

‘Blood cholesterol is still on the rise in the general population of developing countries like Malaysia,’ he continued. ‘This study investigated trends in cholesterol levels in Malaysian patients with acute coronary syndromes.’

The study included 48 851 patients who had an acute coronary syndrome between 2006 and 2015 in Malaysia and were enrolled in the National Cardiovascular Disease Database Acute Coronary Syndrome (NCVD-ACS) registry. This ongoing registry is maintained by the National Heart Association Malaysia with the support of the Ministry of Health Malaysia. Total cholesterol was assessed on entry to the registry.

The researchers examined trends in cholesterol levels of ACS patients over the 10-year period and compared them to previously published values for the entire population. They found a significant trend for declining total cholesterol from 2006 to 2015 in the ACS population (p = 0.012). This was opposite to the total cholesterol trend in the Malaysian population.

ACS patients with a history of coronary heart disease had almost twice the declining rate in cholesterol as those with no history of coronary heart disease. When the researchers examined total cholesterol by type of ACS, they found that patients with unstable angina had the lowest total cholesterol level but the steepest rate of decline, followed by patients with non-ST-elevation myocardial infarction and then patients with ST-elevation myocardial infarction.

Dr Kasim said: ‘We found that blood cholesterol levels have been falling in Malaysian patients with acute coronary syndromes, which is the opposite of the national trend.’

‘Since cholesterol levels have increased significantly in the Malaysian population as a whole, it is highly doubtful that lifestyle change is the reason for the declining cholesterol trend we observed in the ACS population,’ he continued.

Dr Kasim said: ‘While this was an observational study and we cannot infer causality, it seems likely that cholesterol levels decreased as a result of lipid-lowering medication such as statins. ACS patients with a history of coronary heart disease, who were more likely to be taking statins, had a more rapid decline in cholesterol levels than those without a history of coronary heart disease.’

He concluded: ‘These results appear to mimic findings from developed countries in previous years and show that the Malaysian population is reaching similar health milestones. The findings also highlight the need to increase awareness of the harm of raised lipid values and the treatment available.’ Dr Ezam Emran, scientific chair of AFCC 2017, said: ‘This large study suggests that statins are being effectively used by heart attack patients in Malaysia. Rising lipid levels in the general population need to be tackled by promoting healthier lifestyles.’

Professor Michel Komajda, a past president of the ESC and course director of the ESC programme in Brunei, said: ‘The benefits of statins for preventing a second heart attack are unequivocal, as highlighted by the 2017 ESC guidelines. Patients should also be encouraged to quit smoking, adopt a healthier diet and be physically active.’
